# Gyrate atrophy of the choroid and retina: a tertiary center experience

**DOI:** 10.1186/s13023-026-04265-x

**Published:** 2026-02-16

**Authors:** Ekin Özsaydı Aktaşoğlu, Hüseyin Baran Özdemir, Mehmet Cüneyt Özmen, Aslı İnci, İlyas Okur, Fatih Süheyl Ezgü, Leyla Tümer

**Affiliations:** 1 Faculty of Medicine, Department of Pediatrics, Division of Metabolism and Nutrition, Gazi University, Ankara, Turkey; 2 Faculty of Medicine, Department of Ophthalmology, Gazi University, Ankara, Turkey; 3 Department of Pediatrics, Division of Metabolism and Nutrition, University of Health Sciences, Dr. Sami Ulus Child Health Training and Research Hospital, Ankara, Turkey

**Keywords:** Arginine-restricted diet, Hyperornithinemia, Hyperammonemia, Inherited retinal disorders, Pyridoxine treatment

## Abstract

**Background:**

Gyrate atrophy of the choroid and retina (GACR) is a rare amino acid metabolism disorder. Night blindness, cataracts, vision loss, and impaired cognitive functions can be seen. An arginine-restricted diet, combined with pyridoxine, lysine, proline, or creatine supplementation, can be used in conjunction with treatments for ophthalmological findings.

**Methods:**

Patients followed by the Gazi University Faculty of Medicine, Department of Pediatric Metabolism and Nutrition, and Department of Ophthalmology who were genetically or biochemically diagnosed with GACR were included in the study. The patients’ medical records were retrospectively reviewed between 2000 and 2023.

**Results:**

Seven patients with a mean age of 18.08 ± 8.6 years and a mean age at diagnosis of 10.4 ± 5.6 years were included. The earliest diagnosed patient was 15 months old. All patients had high plasma ornithine levels at the time of diagnosis, and they were all started on an arginine-restricted diet. Ophthalmological treatments were decided on a patient-specific basis.

**Conclusions:**

GACR is easily recognizable, but there is still no consensus on treatment modalities. The patients primarily present with progressive ophthalmological findings. An arginine-restricted diet is combined with supplementation with pyridoxine, lysine, proline, or creatine to improve metabolic control. Ophthalmological treatments are mainly applied to reduce cystoid macular edema. Early diagnosis and early initiation of therapy should be aimed at patients, and a multidisciplinary approach should be demonstrated both in diagnosis and follow-up.

## Background

Gyrate atrophy of the choroid and retina (GACR) (OMIM #258870) is a rare autosomal recessive disorder of amino acid metabolism caused by the pathogenic variants of the *OAT* gene [1]. The incidence of the disease is 1:1,500,000 [2], and 70 pathogenic mutations have been identified [3]. *OAT* encodes the pyridoxal-phosphate dependent [4–6] ornithine aminotransferase (OAT) enzymes, which catalyze the transamination of ornithine and α-ketoglutarate to pyrroline-5’-carboxylate and glutamate [4, 6]. A deficiency of OAT causes a slowly progressive chorioretinal degeneration that results in blindness [7]. Myopia and night blindness are common symptoms that patients present with [8], but newborns may present with hyperammonemia and related symptoms [9]. Wang et al. showed that in neonatal mice, the OAT reaction proceeds toward ornithine synthesis, whereas outside the neonatal period, the enzyme catalyzes ornithine degradation [10]. Therefore, OAT deficiency can cause hyperammonemia due to urea cycle damage because of the decreased ornithine synthesis [9]. Due to OAT deficiency in adulthood, ornithine levels increase in body fluids such as plasma, urine, and aqueous humor [9, 11]. High ornithine levels are thought to cause damage to the retinal pigment epithelium [12]; however, there are no ophthalmological findings in Hyperornithinemia–Hyperammonemia–Homocitrullinuria (HHH) syndrome, which detects high ornithine levels [13]. Therefore, the disease’s pathophysiology remains unclear [11].

In addition to ophthalmological symptoms such as night blindness, myopia, cataracts, and vision loss in patients [1], cognitive functions may be impaired due to secondary creatine deficiency due to hyperornithinemia [14–16]. There is no satisfactory treatment, but an arginine-restricted diet in combination with pyridoxine, lysine, proline, or creatine supplementation may be beneficial [1]. With the increasing interest in gene therapies in recent years, it has been demonstrated that AAV-mediated liver-directed gene transfer of OAT, applied to mouse models with GACR, results in a decrease in ornithine levels and an improvement in retinal structure [17].

This study retrospectively examined and reported the demographic and clinical characteristics, ophthalmological findings, and treatment modalities of patients with GACR. Since GACR primarily presents with ophthalmological findings, the importance of follow-up and treatment in cooperation with the ophthalmology department is highlighted.

## Methods

Patients who were followed by the Gazi University Faculty of Medicine, Departments of Pediatric Metabolism and Nutrition and Ophthalmology, and who were genetically or biochemically diagnosed with GACR, were included in this study. The patients’ medical records were retrospectively reviewed between 2000 and 2023. Patients’ demographic information, clinical conditions, treatment adherence, comorbidities, medications, and dietary status were collected. Additionally, ophthalmological findings, including symptoms, medications, and surgical status, were documented in the ophthalmology department’s healthcare records. Fundus images, fluorescein angiography, and fundus autofluorescence images are obtained by the Optos wide-field imaging device (Optos, Inc., USA). Optical coherence tomography, fundus autofluorescence, and fundus angiography images are obtained by the Heidelberg Spectralis device (Heidelberg Engineering GmbH, Heidelberg, Germany). The study was planned in accordance with the principles of the Declaration of Helsinki and was approved by the Gazi University Ethics Commission (No: 2023 − 1523).

## Results

### Demographic data and clinical presentation

Seven patients (P), five male and two female, from five different families born of consanguineous marriages, with a diagnosis of GACR, were included in the study. The data of these patients were evaluated retrospectively. The mean age of the patients was 18.08 ± 8.6 years, and the median age was 16.6 years. Except for one patient (P2), all patients were diagnosed before the age of 18. The mean age at diagnosis was 10.4 ± 5.6 years, and the median was 10.8 years. The earliest diagnosed patient was 15 months old. He was diagnosed in the family screening conducted after his brother (P4) was diagnosed. Patients came in for check-ups every 3–6 months; currently, 3 out of 7 were over 18. The mean age of the patients at the last follow-up was 15 ± 7.02 years, and the median age was 15.5 years.

**Fig. 1:**
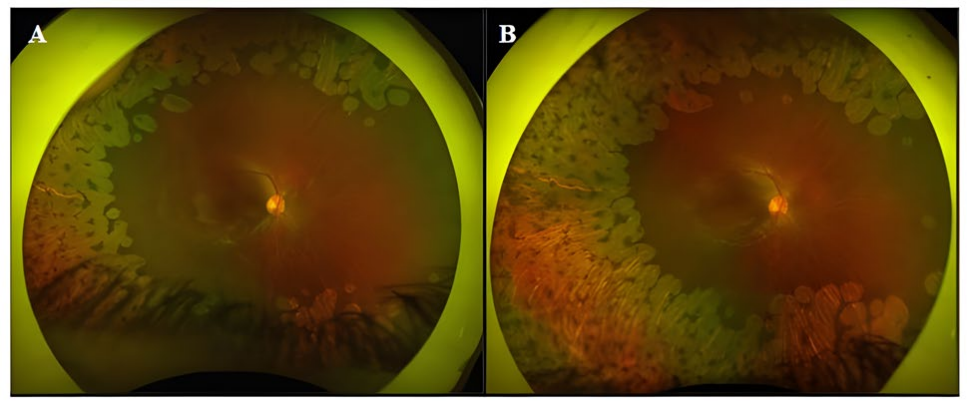
Ultra-widefield color fundus photographs of the right eye of an 8-year-old girl (P5) with gyrate atrophy, taken 3 years apart. (**A**) Well-demarcated patches of chorioretinal atrophy in the mid-periphery, sparing the macula, with visible choroidal vessels underneath and hyperpigmented clumps were present at the initial examination. (**B**) After 3 years, the color fundus photograph showed progression of chorioretinal atrophy

Five of the seven patients were referred from the ophthalmology department to the metabolic outpatient clinic with a preliminary diagnosis of gyrate atrophy. Two of the patients were diagnosed through family screening performed after their brother (P4) was diagnosed. Systemic examination of 6/7 patients was normal; only one patient’s (P7) heart apex was on the right side, and there was a murmur over the mesocardiac focus. From the patient’s history, it was learned that he was being followed up due to dextrocardia. Most patients retrospectively reported progressive nyctalopia and myopia prior to the onset of significant visual loss. At the time of diagnosis, the presenting ophthalmic symptom in 6 of 7 patients was reduced visual acuity. In contrast, the youngest patient (15 months old) was diagnosed through family screening and could not express visual complaints. Two patients (P1 and P2) had lens opacities compatible with cataract, and one patient (P2) had no light perception in the right eye at the last examination, who underwent vitreoretinal surgery due to rhegmatogenous retinal detachment. Retinal findings included sharply demarcated areas of chorioretinal atrophy predominantly in the midperiphery, progressing toward the posterior pole with well-preserved macular islands in earlier stages. Fundus progression of P5 is shown in Fig. 1 as an example. On OCT, marked outer retinal thinning and cystoid macular edema (CME) were observed in five patients (Table 2). Hyperreflective foci in the ganglion cell layer, as previously described in gyrate atrophy by Sen et al. [18], were observed in three patients (42.8%). Overall, no sustained improvement in visual acuity or CME was achieved. The ophthalmological findings of the patients are summarized in Table 1.

**Table 1:** Clinical characteristics of the patients

Patient No	Plasma Ornithine Level at Diagnosis	Hyperammonemia	Treatment			Ophthalmologic Treatment	Comorbid Condition	Mutation Analysis (OAT gene)
			Diet Status	Medications	Pyridoxine Response			
**1**	326.8 mikromol/l (Normal Range: 20–84)	No data	Arginine-restricted diet	No data	No data	Topical Nepefenac for cystoid macular edema	Epilepsy, hypothyroidism	No data
**2**	956.2 mikromol/l (Normal Range: 27–98)	No hyperammonemia	Arginine-restricted diet	Creatine monohydrate, Lysine	No	Vitreoretinal surgery for rhegmatogenous retinal detachment in the right eye, intravitreal aflibercept injection in the left eye due to cystoid macular edema*	Depression	No data
**3**	1118,9 mikromol/ml (Normal Range: 22–97)	No hyperammonemia	Arginine-restricted diet	Creatine monohydrate	Yes	Topical Nepefenac + Brinzolamide for cystoid macular edema	-	c.991 C > T (p.Arg331Ter) Homozygous, Pathogenic
**4**	872.9 mikromol/l (Normal Range: 20–90)	No hyperammonemia	Arginine-restricted diet	Creatine monohydrate	Yes	Subretinal PRP injection due to cystoid macular edema* + Topical Nepefenac for cystoid macular edema	-	c.424 + 1G > A Homozygous, Likely pathogenic
**5**	1014 mikromol/l (Normal Range: 20–80)	No hyperammonemia	Arginine-restricted diet	Creatine monohydrate	Yes	Topical Coenzyme Q10 &Vitamin E TPGS^¶^ + Subretinal PRP injection due to cystoid macular edema* + Topical Nepefenac for cystoid macular edema*	-	c.424 + 1G > A Homozygous, Likely pathogenic
**6**	514.3 mikromol/l (Normal Range: 9-123)	No hyperammonemia	Arginine-restricted diet	Creatine monohydrate	Yes	Subretinal PRP injection due to cystoid macular edema* + Topical Nepefenac for cystoid macular edema	-	c.424 + 1G > A Homozygous, Likely pathogenic
**7**	1462 umol/l (Normal Range: 20–136)	No data	Arginine-restricted diet	-	Yes	No treatment	Dextrocardia, mild mental retardation	No data

^*^Treatment was discontinued due to lack of response, ^¶^Regression of cystoid macular edema was observed first but the effect did not continued

*PRP: Platelet rich plasma*,* TPGS: D-ɑ-tocopheryl polyethylene glycol succinate*

**Table 2:** Ophthalmological findings of the patients

Patient No	Age at Last Ophtalmic Examinaton (year)	Presenting Ophthalmic Symptoms	Visual Acuity	Presence of Cataract	Presence of Macular Oedema	Baseline CFT (µm)	Final CFT (µm)	CME Response	Hyperreflective Foci at the Ganglion Cell Layer
**1**	19	Nyctalopia	OU: 20/100	OU: PSCC	OU: CME	OD: 502 OS: 509	OD: 297 OS: 466	Partial resolution	Present
**2**	27	Nyctalopia	OD: NLP OS: 20/40	OS: PSCC	OS: CME	OS: 313	OS: 320	Stable	Absent
**3**	16	Nyctalopia and myopia	OD: 20/50 OS: 20/40	No cataract	OU: CME	OD: 204 OS:234	OD: 191 OS: 255	Stable	Absent
**4**	15	Nyctalopia and myopia	OU: 20/100	No cataract	OU: CME	OD: 719 OS: 676	OD: 512 OS: 583	Partial resolution	Present
**5**	10	Nyctalopia and myopia	OU: 20/50	No cataract	OU: CME	OD: 570 OS: 589	OD: 634 OS: 600	Worsened	Present
**6**	4	No symtom	n.a.	No cataract	No CME Peripheral gyrate atrophy	OD: 248 OS: 281	OD: 251 OS: 267	Stable (no CME)	Absent
**7**	12	Loss of vision	n.a.	n.a.	n.a.	n.a.	n.a	n.a	n.a.

CFT: Central Foveal Thickness

OU: oculus uterque, both eyes

OS: oculus sinistra, left eye

OD: oculus dextra, right eye

PSCC: Posterior subcapsular cataract

CME: Cystoid macular edema

NLP: No light perception

n.a.: not available

Fundus images, fluorescein angiography, and fundus autofluorescence images are obtained by optos wide-field imaging device (Optos, Inc., USA)

Optical coherence tomography, fundus autofluorescence and fundus angiography images are obtained by the Heidelberg Spectralis device (Heidelberg Engineering GmbH, Heidelberg, Germany)

One patient (P1) had epilepsy and hypothyroidism in addition to gyrate atrophy, and patient 2 was using a Selective Serotonin Reuptake Inhibitor (SSRI) as a comorbid condition. The patient, who was followed up with dextrocardia (P7), also had mild intellectual disability and was attending a special education program.

### Biochemical and genetic findings

All patients had high plasma ornithine levels at the time of diagnosis. 5/7 patients did not have hyperammonemia. The other two patients did not have clinical findings compatible with hyperammonemia; however, the laboratory ammonia value was not documented in the records (Table 2). Two of the seven patients did not attend regular follow-up visits; however, the other five attended regularly, and all had high plasma ornithine levels at the last check-up. The last control urine ornithine levels of 4/7 patients were found to be high.

Genetic analysis was performed on four of seven patients, and two different, defined homozygous mutations were detected. c.991 C > T (p.Arg331Ter) variant was detected in one patient (P3). The c.424 + 1G > A variant was detected in 3 sibling patients (P4, P5, and P6). c.424 + 1 GA was defined as likely pathogenic according to the American College of Medical Genetics (ACMG). It was a splice donor mutation (NM_000274.4: c.424 + 1G > A) and was previously defined in ClinVar (ClinVar ID: 2843028). c.991 C > T was a nonsense mutation (NM_000274.4: c.991 C > T) and pathogenic according to ACMG and defined before in Clinvar (Clinvar ID: 56141). Both variants have been reported previously [6, 19].

### Treatment

The management of OAT includes restricting dietary arginine, B6 treatment based on responsiveness, and creatine supplementation. Since the OAT enzyme is pyridoxal phosphate-dependent, patients were first assessed for responsiveness to B6 before beginning the arginine-restricted diet. Five patients were evaluated as pyridoxine-responsive and added to their treatment. Patient 2 was unresponsive to pyridoxal phosphate, and the dosage was reduced to the maintenance dose. Due to irregular follow-up visits, the pyridoxal phosphate response could not be evaluated in patient 1. All patients were started on an arginine-restricted diet. The amount of protein the patients consumed was restricted, and their diets were supplemented with low-protein formulas. Creatine monohydrate was added to the treatment of 5/7 patients due to secondary creatine deficiency, and lysine supplementation was added in addition to creatine monohydrate in one patient (P2). Poor compliance with the arginine-restricted diet and medication use was observed in patients.

**Fig. 2:**
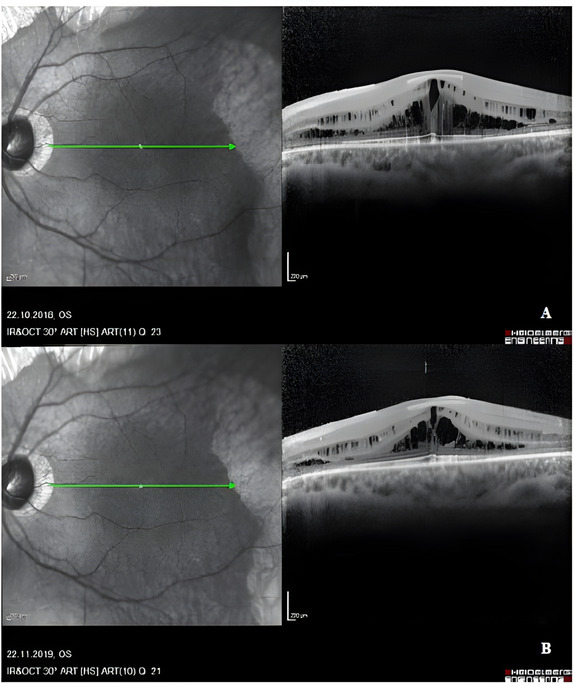
Spectral-domain optical coherence tomography images of the left eye of an 18-year-old male patient (P1) with gyrate atrophy taken 1 year apart. Cystoid macular edema (**A**) seen on initial examination slightly regressed (**B**) after topical nepafanac 4 × 1 and oral acetazolamide 2 × 1

**Fig. 3:**
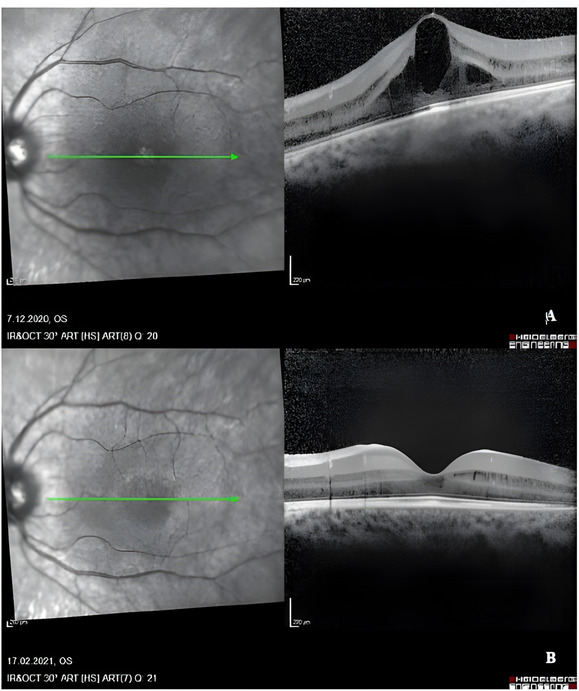
Spectral-domain optical coherence tomography images of the left eye of an 8-year-old female patient (P5) with gyrate atrophy were taken 3 months apart. Cystoid macular edema (**A**) seen on initial examination completely regressed (**B**) after topical Coenzim Q10 &Vitamin E TPGS drops

Since patients’ ophthalmological findings are the primary cause of symptoms, ophthalmologic treatment modalities were added to an arginine-restricted diet and metabolic treatments. Topical nonsteroidal anti-inflammatory drugs (NSAIDs), such as nepafenac, and carbonic anhydrase inhibitors (CAIs), such as brinzolamide, are used in ophthalmology to treat cystoid macular edema (CME). Subtenon PRP has also been tried to treat CME and was attempted in two patients as an experimental approach, but no significant anatomical or functional improvement was observed. Topical nepefenac treatment for CME was attempted in 5/7 patients (Fig. 2); however, no maintained response was observed. Subtenon PRP was attempted in two siblings (P4 and P6) with the same mutation as an experimental approach; however, no significant anatomical or functional improvement was observed. Topical Coenzyme Q10 & Vitamin E D-α-tocopheryl polyethylene glycol succinate (TPGS) was attempted in the other sibling (P5). Although TPGS treatment was beneficial for CME regression (Fig. 3), no response was observed in the follow-up. Only P2 required vitreoretinal surgery due to rhegmatogenous retinal detachment in the right eye during follow-up. There is no data regarding the ophthalmological treatment of P7 because his follow-up was irregular. The treatment status of the patients is summarized in Table 2.

## Discussion

Inherited retinal diseases (IRD) have been associated with more than 300 genes to date (RetNet, Retinal Information Network, https://sph.uth.edu/retnet, accessed on 1 November 2023), and GACR due to an *OAT* gene defect is one of them [20]. This study presented data from seven patients who were followed up in the Gazi University Faculty of Medicine, Pediatric Metabolism and Nutrition, and Ophthalmology departments with GACR. Four cases from our center, published by Hasanoğlu et al. [21] in 1996, were excluded from this study to avoid duplication. It was observed that most of the patients were diagnosed late, and their diet and treatment compliance were poor. It was observed that as the patients’ ophthalmological symptoms worsened, their metabolic check-ups decreased, and two patients even discontinued follow-up.

The relationship between increased plasma ornithine levels and gyrate atrophy was first recognized by Simell and Takki in 1973 [22]. Additionally, in a review by Hayasaka et al. [23], it was mentioned that high ornithine levels or its metabolites are toxic to the retinal pigment epithelium. Studies on gyrate atrophy in the literature are mainly in case reports or case series. The review study by Montioli et al. [2] and the cohort study by Palmer et al. [24], which included 18 patients, are among the most comprehensive publications in the literature. In recent years, with developments in retinal imaging, reviews that guide the recognition of IRD findings have begun to be published [20]. However, the rarity of the disease, differences in treatment and follow-up modalities across studies and centers, and the lack of randomized controlled trials with large patient groups contribute to variation in the treatment and follow-up of patients with GACR.

The most commonly used treatment method is an arginine-restricted diet [1]. In a study by Hayasaka et al., OAT-deficient mice fed an arginine-restricted diet showed better ophthalmological function than those fed a normal diet [23]. On the other hand, there is limited research on whether a protein-restricted diet improves disease progression [1]. In our cohort, poor diet and medication compliance among patients make it challenging to investigate the effects of a protein-restricted diet on disease progression. Studies with long-term follow-up of a more extensive series of GACR patients on a protein-restricted diet, incorporating a multidisciplinary approach, are required, especially for patients diagnosed early.

Pyridoxine is also used in the treatment of GACR patients [25]. Studies show that pyridoxine increases residual enzyme activity or acts as a chaperone. Both pyridoxine-responsive and pyridoxine-unresponsive cases have been reported in the literature, and there is no clear dose response [1]. In the review by Balfoort et al. [1], 30% of patients were found to be pyridoxine-responsive. However, the molecular mechanisms explaining the response of pathogenic variants to pyridoxine have not been adequately elucidated [2]. When L-lysine is used in therapy, plasma lysine concentration increases, and competitive inhibition of arginine and ornithine reabsorption from the kidneys is hypothesized [1]. This hypothesis was tested by Peltola et al., and the addition of oral L-lysine to the treatment of 5 GACR patients who did not respond to pyridoxine resulted in a decrease in plasma ornithine levels and an increase in urinary ornithine excretion [26]. Proline and creatine monohydrate are still used in treatment [1]; however, centers employ different treatment modalities.

In our cohort, electroretinography (ERG) data were unavailable for all patients due to the study’s retrospective design and the long follow-up period. However, previous studies have shown that ERG responses in patients with gyrate atrophy are typically markedly reduced or extinguished at diagnosis, reflecting severe dysfunction of both rod and cone photoreceptors [27, 28]. Long-term metabolic control through dietary and pharmacologic treatment may slow the rate of functional decline as measured by ERG. Still, meaningful improvement in ERG parameters has not been demonstrated once retinal degeneration is established [29]. Therefore, ERG primarily serves as a diagnostic and baseline functional assessment tool rather than a sensitive marker for short-term therapeutic efficacy in GACR.

The most obvious findings of the disease are ophthalmological problems; however, in a study by Palmer et al. involving 13 individuals, many comorbid conditions accompanied by developmental delay (6/13) were among the most common [24]. Also, reduced fat-free mass (9/13), hematological, respiratory, endocrinological, and urological conditions were also stated [24]. In our cohort, epilepsy (P1), hypothyroidism (P1), depression (P2), dextrocardia, and mild intellectual disability (P7) were detected as comorbidities. Since the pathogenesis of the disease is not fully understood, it is challenging to determine whether the current findings reflect a comorbid condition or systemic involvement of GACR.

NSAIDs such as nepafenac and bromfenac have been widely used in ophthalmic conditions associated with IRD, often in combination with carbonic anhydrase inhibitors (CAIs) to manage CME [30, 31]. In gyrate atrophy, topical NSAIDs have shown some effectiveness in reducing retinal edema when combined with other treatments, such as CAIs, as indicated by case reports showing improvement in macular edema with combined topical therapies [32]. Five of seven patients in our cohort received topical nepafenac, but responses were generally unsatisfactory. This aligns with the limited success reported in the literature, where NSAIDs have shown inconsistent effects in treating macular edema associated with IRD. While NSAIDs are often utilized due to their anti-inflammatory properties, their limited efficacy in GACR underscores the need for alternative approaches or combination therapies to manage CME in these patients better. Further studies are required to evaluate their role in the long-term management of ophthalmological complications in GACR.

The limited use of CAIs in our cohort is attributed to their variable and often inconsistent efficacy in managing CME in IRD. Previous studies have demonstrated that while CAIs, such as acetazolamide and brinzolamide, may reduce CME in retinitis pigmentosa and similar IRDs, their effectiveness in gyrate atrophy of the choroid and retina (GACR) is less conclusive [31]. In our cohort, CAIs were initiated in a subset of patients, but the overall response was limited, consistent with the literature, which reports inconsistent results. Therefore, the decision to use CAIs was patient-specific and based on individual responses to previous treatment interventions. This selective application reflects the clinical reality that CAIs may not universally benefit all patients with GACR and underscores the need for alternative therapeutic strategies.

PRP, rich in growth factors, has been explored in various retinal pathologies, such as retinitis pigmentosa [33]. However, the available evidence is limited and largely based on small, heterogeneous studies, and its role in cystoid macular edema remains unproven. To date, no clinical studies have specifically evaluated the use of subtenon PRP in GACR. In the present study, subtenon PRP was attempted experimentally; however, no significant anatomical or functional improvement in macular edema was observed.

Topical Coenzyme Q10 (CoQ10) combined with Vitamin E TPGS has shown promising effects in retinal and neuroprotective treatments due to its antioxidant properties and ability to enhance bioavailability. CoQ10 plays a critical role in mitochondrial energy production and protection against oxidative stress, while TPGS, a water-soluble derivative of Vitamin E, improves the permeability and efficacy of lipophilic compounds like CoQ10. Topical Coenzyme Q10 (CoQ10) combined with Vitamin E TPGS has demonstrated neuroprotective and antioxidant effects primarily in non-human experimental models, including rodent and mouse models of ocular hypertension, optic nerve injury, and retinal damage [34–36]. These studies have shown reduced retinal ganglion cell apoptosis and preservation of retinal structure. Clinical evidence in humans remains limited and has largely been restricted to small studies of glaucoma patients and ocular surface conditions, rather than inherited retinal diseases or cystoid macular edema. Therefore, any potential benefit of CoQ10–TPGS in GACR remains speculative. In our cohort, topical CoQ10–TPGS was associated with a transient reduction in macular edema in one patient; however, this effect was not sustained during follow-up. Further controlled clinical studies are required before its use can be recommended in GACR.

This study has some limitations inherent to its retrospective design, including the lack of uniform documentation of ophthalmological and metabolic parameters before and after treatment. Because patients were followed for an extended period and diagnostic modalities evolved, complete pre- and post-treatment data were not available for all cases. Despite these limitations, the study provides valuable insights into real-world management and long-term outcomes of patients with gyrate atrophy.

In conclusion, although GACR is readily recognized by characteristic ophthalmological findings, its poorly understood pathophysiology leaves treatment options unclear, and there remains no consensus on treatment. The patients primarily present with ophthalmological findings, and despite metabolic and ophthalmological treatments, their conditions often progress. Therefore, it is essential to diagnose GACR as early as possible to initiate an arginine-restricted diet and manage other metabolic parameters, in addition to ophthalmologic treatment. This study addresses patients from a metabolic perspective and emphasizes the importance of metabolic follow-up alongside ophthalmological treatments. The aim should be to achieve symptom-free living and improve quality of life through therapy in collaboration with the departments of metabolism and ophthalmology.

## Data Availability

Data will be available on a reasonable request.
